# Supplementary material for: The Molecular and Function Characterization of Porcine MID2
Source: Animals (Basel). 2023 Sep 8;13(18):2853. doi: 10.3390/ani13182853 (PMC10526110; doi:10.3390/ani13182853)
Supplement: Supplementary file 1 [file animals-13-02853-s001.zip › animals-2561879-supplementary.pdf]

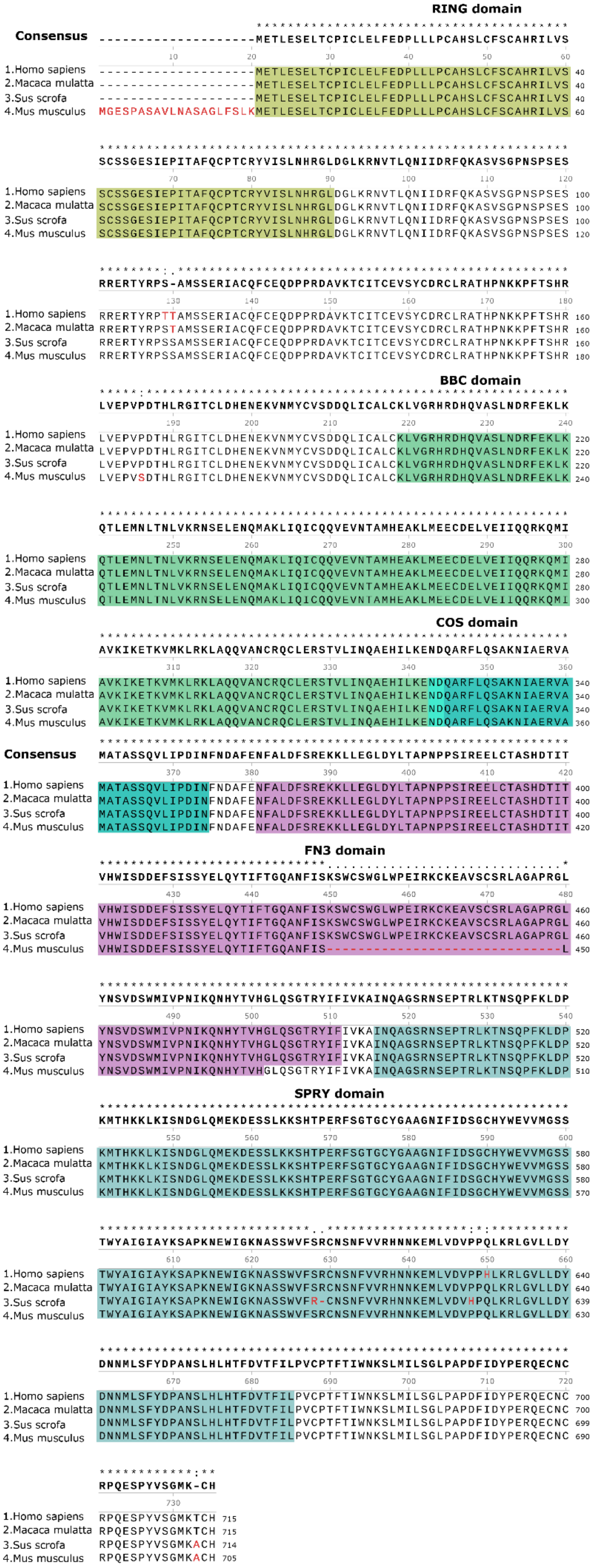
RING domain
Consensus
1.Homo sapiens
2.Macaca mulatta
3.Sus scrofa
4.Mus musculus
BBC domain
COS domain
FN3 domain
SPRY domain

Figure S1. Alignment of the deduced pMID2 (Sus scrofa) amino acid sequences with Homo sapiens, Macaca mulatta and Mus musculus. The amino acid sequences were aligned using CLUSTALW2.1 (http://www.genome.jp/tools-bin/clustalw). The RING domain is highlighted in slightly desaturated yellow, BBC domain in lime green, COS domain in moderate cyan, FN3 domain in slightly desaturated magenta and SPRY domain in slightly desaturated cyan. Asterisks (*) indicate identical or conserved residues; colons (:) indicate conserved substitutions; dots (.) indicate semi-conserved substitutions in the multiple sequence alignment. Dashes (-) indicate gaps in the alignment.
